# Supplementary material for: Tanycytes control the hormonal output of the hypothalamic-pituitary-thyroid axis
Source: Nat Commun. 2017 Sep 7;8:484. doi: 10.1038/s41467-017-00604-6 (PMC5589884; doi:10.1038/s41467-017-00604-6)
Supplement: Supplementary file 1 — Supplementary Information [file 41467_2017_604_MOESM1_ESM.pdf]

File Name: Supplementary Information
Description: Supplementary Figures and Supplementary Table.

File Name: Supplementary Movie 1
Description: $[Ca^{2+}]_i$ increase in tanycytes of the ME after bath application of taltirelin (33 µM), 2 weeks after transduction of C57Bl/6 mice with the calcium sensor GCamP6s. Real time: 5 min, 1 picture s^{-1} .

File Name: Supplementary Movie 2
Description: $[Ca^{2+}]_i$ increase in tanycytes of the ME after bath application of taltirelin (33 µM) and coapplication of midazolam (500 µM), 2 weeks after transduction of C57Bl/6 mice with the calcium sensor GCamP6s. Real time: 5 min, 1 picture s^{-1}.

File Name: Supplementary Movie 3
Description: $[Ca^{2+}]_i$ increase in tanycytes of the ME after bath application of taltirelin (33 µM) and 20 min after washout of midazolam (500 µM). Two weeks before C57Bl/6 mice were transduced with a rAAV vector expressing the calcium sensor GCamP6s. Real time: 5 min, 1 picture s^{-1}.

File Name: Supplementary Movie 4
Description: 3D-reconstruction of tanycytes (red) of the lateral part of the ME of Tan3D mice, two weeks after tamoxifen injection. mCherry detection was increased with immunohistochemistry. Blue, DAPI. For 3D reconstruction the Imaris software was used.

File Name: Peer Review File

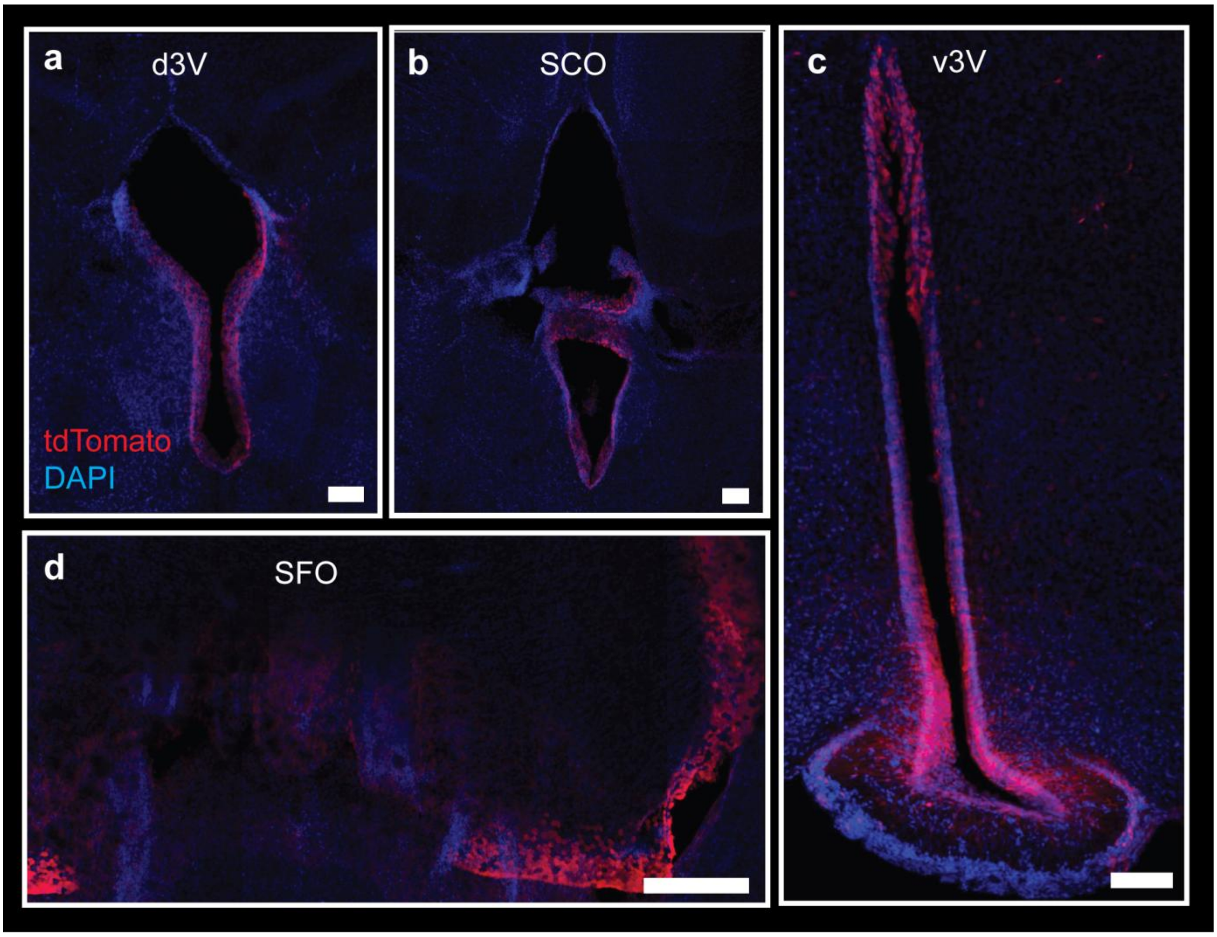

Supplementary Figure 1: Transduction profile of AAV-CMV-iCre-2A-GFP in Ai14 reporter mice. (a-d) Representative confocal images of circumventricular areas of the brain of Ai14 mice 2 weeks after injection of AAV-CMV-iCre-2A-GFP (5x10^9 genomic particles) in the lateral ventricle. **(a)** Dorsal part of 3rd ventricle (d3V), **(b)** subcommissural organ (SCO), **(c)** ventral part of 3rd ventricle (v3V), **(d)** subfornical organ (SFO). Scale bar, 100 µm; red, tdTomato-positive cells; blue, DAPI.

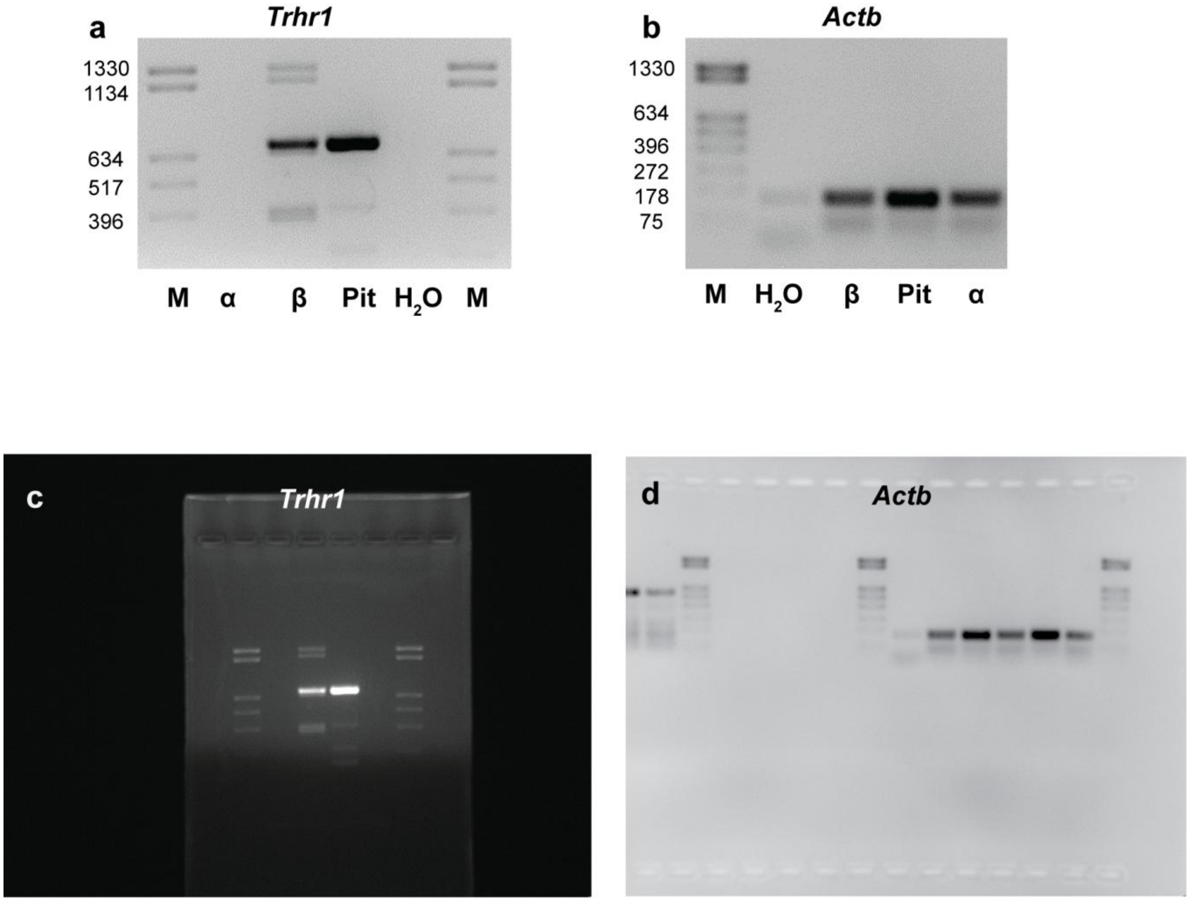

Supplementary Figure 2: Expression of *Trhr1* mRNA in tanycytic subpopulations. RT-PCR detection of *Trhr1* mRNA (**a**, size: 693 bp) and actin (**b**, *Actb*, size: 140 bp) from microdissected samples of α- and β-tanycytes. cDNA from pituitary (Pit) was used as positive and H_2O as negative control. M, marker. **(c and d)** Original pictures of the agarose gels.

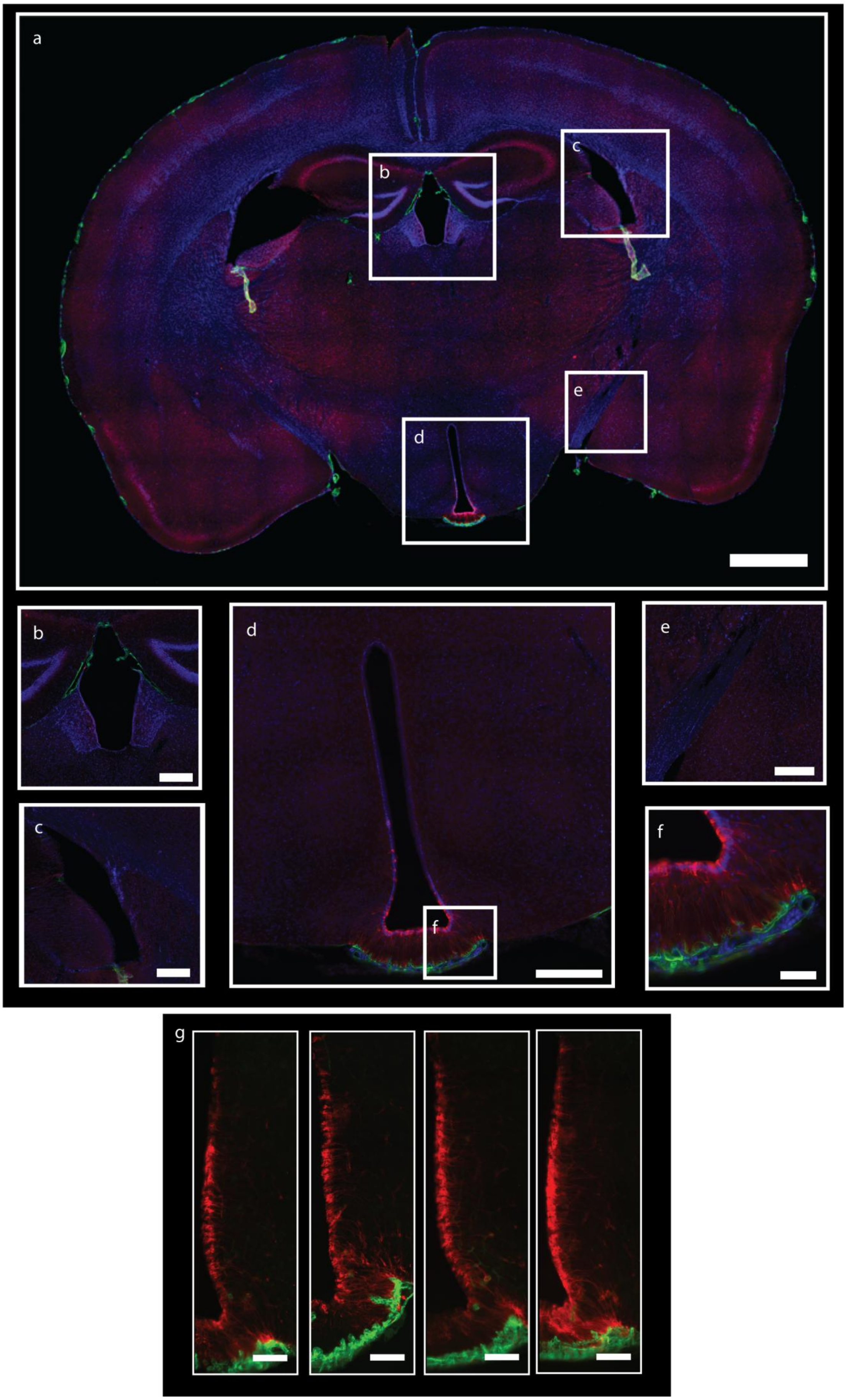

Supplementary Figure 3

Supplementary Figure 3: Transduction profile of AAV-CAG-flex(hM3D-mCherry) in *GlastCreER*^{T2} mice. Immunostaining for hM3D-mCherry (red) and collagen IV (green) in the brain of *GlastCreER*^{T2} mice transduced with AAV-CAG-flex(hM3D-mCherry) (gTan^{3D}). Stainings were performed 2 weeks after tamoxifen administration. **(a)** Whole coronary brain section (Bregma -1,8 mm; scale bar, 1 mm; blue, DAPI). Close-ups in the dorsal part of 3rd ventricle **(b)**, lateral ventricle **(c)**, ventral part of 3rd ventricle **(d)**, and optical tract **(e)**. Scale bar, 250 µm. **(f)** Close-up of the ventral 3rd ventricle in the area of the ME. Scale bar, 50 µm. **(g)** Representative pictures of the recombination of hM3D-mCherry in the ventral 3rd ventricle of four gTan^{3D} animals (red, mCherry; green, collagen IV; scale bar, 100 µm). Microscopic settings for collagen IV visualization were optimized for the strong staining in the ME.

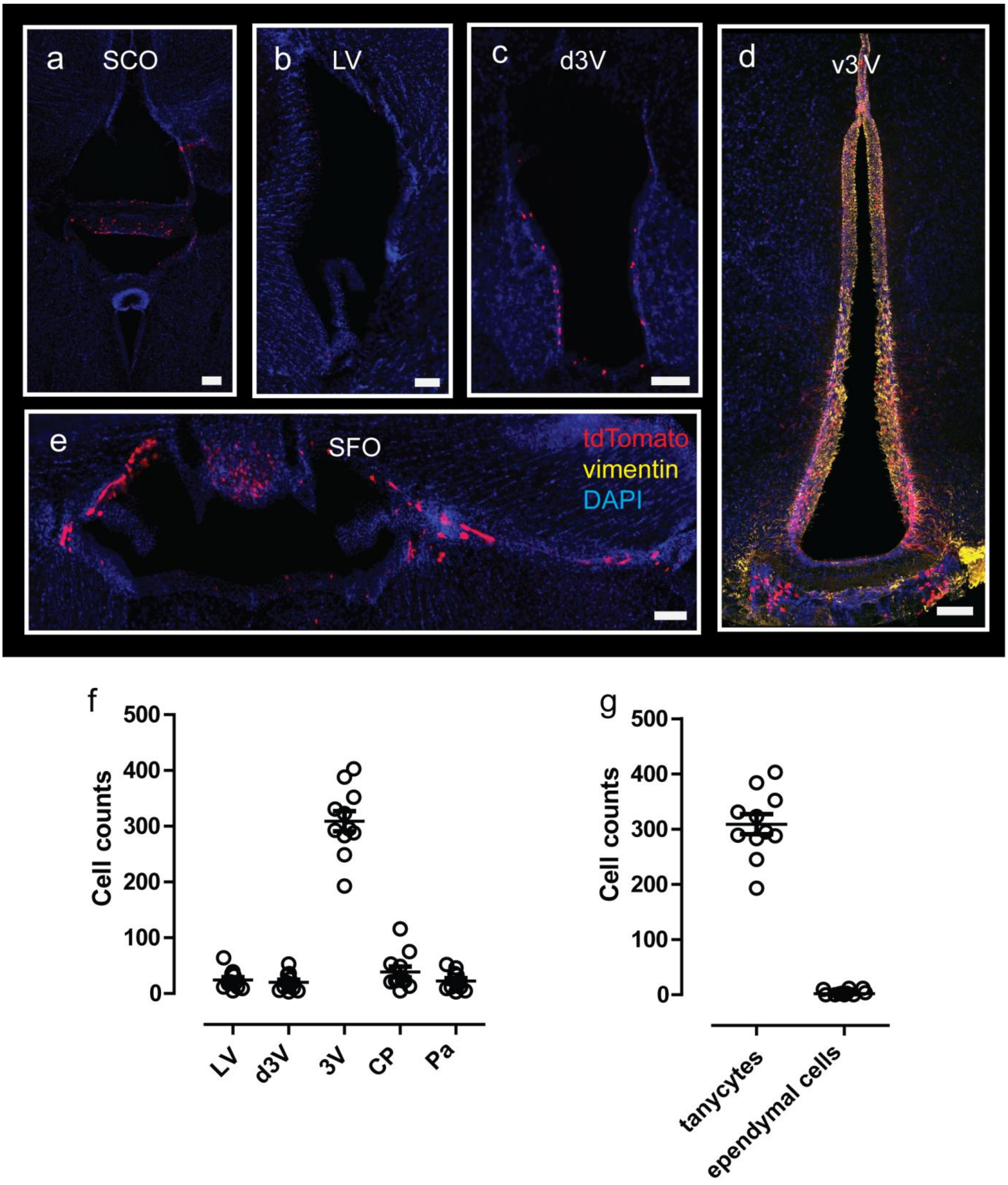

Supplementary Figure 4: Transduction profile of AAV-Dio2-iCre-2A-GFP. (**a-e**) Representative confocal images of circumventricular areas of the brain of Ai14 reporter mice 2 weeks after injection of AAV-Dio2-iCre-2-GFP (5x10^9 genomic particles) in the lateral ventricle (LV). SCO, subcommissural organ; d3V, dorsal part of 3rd ventricle; v3V, ventral part of 3rd ventricle; and SFO, subfornical organ. Scale bar, 100 µm; red, tdTomato; yellow, vimentin; blue, DAPI. **(f)** Distribution of hM3D-mCherry expressing cells in various brain

areas after mice were injected with AAV-CAG-flex(hM3D-mCherry) plus AAV-Dio2-iCre-2A-GFP ($2x10^{10}$ genomic particles of each vector, 2.5 µl). In the indicated brain areas, we counted hM3D-mCherry-positive cells on 3 consecutive coronal brain sections (50-µm thick), corresponding to Bregma -1.8 to -1.95 mm. Values are cell counts per animal and anatomical area or means±S.E.M (n=11 animals). CP, choroid plexus; Pa, parenchyma. **(g)** Further differentiation of cells in v3V showed that mainly tanycytes and not ependymal cells were transduced. Tanycytes and ependymal cells were differentiated based on the localization and cell morphology.

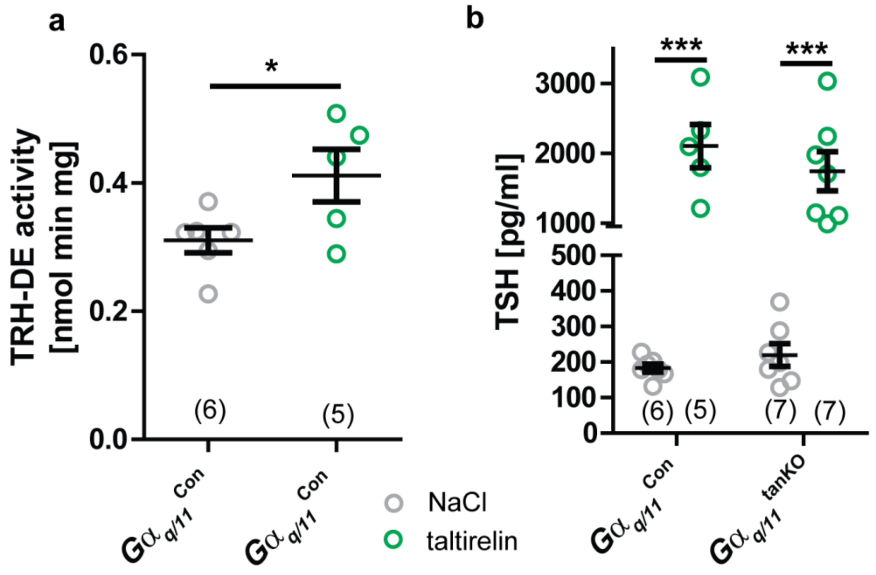

Supplementary Figure 5: Effects of the TRH receptor agonist taltirelin on TRH-DE and TSH plasma concentrations. (a) TRH-DE activity in $G\alpha_{q/11}{}^{Con}$ mice 2 hours after stimulation with saline (gray; NaCl, 0.9%) or taltirelin (green; 1 mg kg^{-1} body weight; i.p.); *p=0.0427 (two-tailed Student's t-test); mean±S.E.M.; n, as indicated **(b)** TSH plasma concentrations 2 hours after stimulating $G\alpha_{q/11}{}^{Con}$ and $G\alpha_{q/11}{}^{tanKO}$ mice with saline (NaCl, 0.9%; gray) or taltirelin (green; 1 mg kg^{-1} body weight; i.p.). Two-way ANOVA; $F(1/22)_{taltireline}=78.15$, $p<0.0001$; $F(1/22)_{genotype}=0.7$, $p=0.41$; $F(1/22)_{interaction}=1.1$, $p=0.32$; ***p<0.0001 between indicated groups (Bonferroni post-test); mean±S.E.M; n, as indicated.

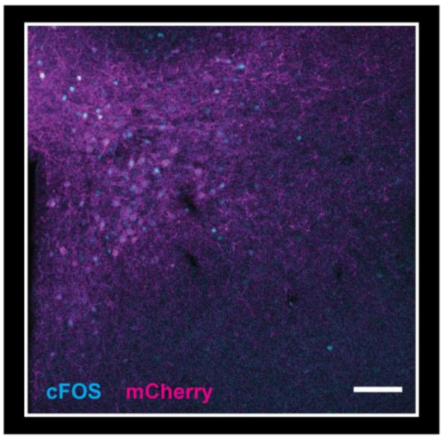

Supplementary Figure 6: Activation of cFos in AAV-TRH-hM3D-mCherry transduced neurons of the PVN after CNO stimulation. Costaining of hM3D-mCherry (magenta) and cFos (cyan) in the PVN 45 min after CNO administration and 2 weeks after bilateral injection of AAV-TRH-hM3D-mCherry in the PVN of C57Bl/6 mice (PVN^{3D}-Bl6; scale bar, 100 µm).

Supplementary Table 1: Summary of the mouse models generated in this study. LV, lateral ventricle; PVN, paraventricular nucleus; C57Bl/6, wild-type strain.

	**AAV (site of injection)**	**Genotype of mice**	**Short name of mice**	**Purpose/Effect**
Supplementary Fig. 1	AAV-CAG-Cre (LV)	Ai14 Cre reporter	Ai14	Expression of tdTomato in tanycytes
Fig. 1	AAV-CAG-GCamP6s (LV)	C57Bl/6	Bl6	Expression of GCamP6s in tanycytes
Fig. 2	AAV-CAG-GCamP6s (LV)	C57Bl/6	Bl6	Expression of GCamP6s in tanycytes of C57Bl/6 mice
		$Trhr1^{-/-}$	$Trhr1^{-/-}$	Expression of GCamP6s in tanycytes of $Trhr1^{-/-}$ mice
		$Trhr2^{-/-}$	$Trhr2^{-/-}$	Expression of GCamP6s in tanycytes of mice $Trhr2^{-/-}$ mice
		$GlastCreER^{T2}$; $G\alpha_{11}^{-/-}G\alpha_{q}^{fl/fl}$	$G\alpha_{q/11}^{gliaKO}$	Expression of GCamP6s in tanycytes of mice with deficiency of $G\alpha_{q/11}$ proteins in glia and tanycytes
Fig. 3, Supplementary Fig. 3	AAV-CAG-flex(hM3D-mCherry) (LV)	$GlastCreER^{T2}$	$gTan^{3D}$	Selective expression of hM3D-mCherry in tanycytes
Fig. 4a, b, Supplementary Fig. 4a-e	AAV-Dio2-iCre-2A-GFP (LV)	Ai14 Cre reporter	Ai14	Selective expression of tdTomato in tanycytes
Fig. 4c-g, Supplementary Fig. 5	AAV-Dio2-GFP (LV)	$G\alpha_{11}^{-/+}G\alpha_{q}^{fl/fl}$	$G\alpha_{q/11}^{Con}$	Littermate control for $G\alpha_{q/11}^{tanKO}$
	AAV-Dio2-iCre-2A-GFP (LV)	$G\alpha_{11}^{-/-}G\alpha_{q}^{fl/fl}$	$G\alpha_{q/11}^{tanKO}$	Selective deletion of $G\alpha_{q/11}$ proteins in tanycytes
Fig. 5, Supplementary Fig. 4f and g	AAV-Dio2-iCre-2A-GFP (LV) + AAV-CAG-flex(hM3D-mCherry) (LV)	C57Bl/6	$dTan^{3D}$-Bl6	Tanycyte selective expression of hM3D-mCherry in C57Bl/6 mice
		$G\alpha_{11}^{-/-}G\alpha_{q}^{fl/fl}$	$dTan^{3D}$-$G\alpha_{q/11}^{tanKO}$	Tanycyte selective expression of hM3D-mCherry and deletion of $G\alpha_{q/11}$
Fig. 6, Supplementary Fig. 6	-	C57Bl/6	PVN^{Con}-Bl6	Chemogenetic stimulation of TRH neurons in C57Bl/6 mice
	AAV-TRH-hM3D-mCherry (PVN)		PVN^{3D}-Bl6	
	-	$Trhr1^{-/-}$	PVN^{Con}-$Trhr1^{-/-}$	Chemogenetic stimulation of TRH neurons in $Trhr1^{-/-}$ mice
	AAV-TRH-hM3D-mCherry (PVN)		PVN^{3D}-$Trhr1^{-/-}$	
Fig. 7	AAV-Dio2-GFP (LV)	$G\alpha_{11}^{-/+}G\alpha_{q}^{fl/fl}$	PVN^{Con}-$G\alpha_{q/11}^{Con}$	Controls for PVN^{3D}- $G\alpha_{q/11}^{Con}$
	AAV-Dio2-GFP (LV) + AAV-TRH-hM3D-mCherry (PVN)	$G\alpha_{11}^{-/+}G\alpha_{q}^{fl/fl}$	PVN^{3D}-$G\alpha_{q/11}^{Con}$	Chemogenetic stimulation of TRH neurons in control mice
	AAV-Dio2-iCre-2A-GFP (LV) + AAV-TRH-hM3D-mCherry (PVN)	$G\alpha_{11}^{-/-}G\alpha_{q}^{fl/fl}$	PVN^{3D}-$G\alpha_{q/11}^{tanKO}$	Chemogenetic stimulation of TRH neurons plus selective deletion of $G\alpha_{q/11}$ proteins in tanycytes
